# Competitiveness of citrus export and its determinants: a two-way fixed effect panel data model approach

**DOI:** 10.3389/fnut.2024.1414478

**Published:** 2024-06-10

**Authors:** Naveed Hayat, Muhammad Naeem, Ghulam Mustafa, Bader Alhafi Alotaibi, Abou Traore, Aftab Anwar

**Affiliations:** ^1^Department of Economics, Division of Management and Administrative Science, University of Education, Lahore, Pakistan; ^2^Department of Agricultural Extension and Rural Society, King Saud University, Riyadh, Saudi Arabia; ^3^Department of Community Sustainability, College of Agricultural & Natural Resources, Michigan State University, East Lansing, MI, United States

**Keywords:** citrus exports, cross-border factors, Pakistan, panel data, gravity model, two-way fixed effect regression

## Abstract

Pakistan has a conducive condition for the development of a wide range of scrumptious fruits. As a result, the country grows a diverse assortment of tropical and subtropical fruits; the most prized and top-ranked fruit among all fruits grown in Pakistan is citrus. Citrus is the principal fruit that contributes significantly to Pakistan’s export earnings and national income. In this study, the cross-border determinants influencing Pakistan’s citrus exports to its topmost 22 trading partners are examined using a gravity model technique. This is the first large study from Pakistan by using gravity model to check the impact of various cross-border factors on citrus fruit export. The analysis is based on a panel dataset covering the years 2003 to 2021. To estimate the results, the study used fixed effect regression with time and country fixed effects. The results signify that *per capita* income, population, and some regional dummies are positively associated with citrus exports from Pakistan. Citrus price, distance, exchange rate, and other regional dummies are observed to have an adverse effect on citrus exports. Trade agreements between Pakistan and trade partners such as free trade agreements, preferential trade agreements, and SAFTA, have been observed as important determinants of citrus exports. Citrus exporters in Pakistan can also benefit from understanding the factors that influence export markets. By addressing the challenges identified in this study, Pakistan can enhance its citrus exports and boost its agricultural sector.

## 1 Introduction

Nature has endowed Pakistan with an ideal climate for the cultivation of a broad variety of scrumptious fruits. As a result, the country grows a diverse assortment of tropical and subtropical fruits. Pakistanis scientists have created unique strains of exotic fruit varietals throughout the years. Fruit production is an essential sub-sector of agriculture that plays a crucial role not only in the revitalization of the rural economy but also in improving human nutrition, which is frequently insufficient in elements such as vitamins and minerals. The primary fruit crops that contribute significantly to national income are citrus and mango. Citrus is a treasured fruit in Pakistan, and it ranks first in both cultivation area and production volume among all fruits. Citrus fruits, such as oranges, grapefruits, and lemons, originated in the tropical regions of the Southern Himalayas, Southeast Asia, and the Indonesian Archipelago. Over time, they spread to many different parts of the world, growing in a belt that extends 35 degrees north and south of the equator. This citrus belt includes countries such as Pakistan, India, China, the United States, Brazil, and South Africa. The range of qualities in citrus fruit varies according to geography. Semi-tropical climatic zones around the southern and northern latitude boundaries are ideal for economic agriculture (1).

Originally Kinnow, most delectable citrus variety, was developed by Citrus Research Center of the University of California, Riverside, USA. It was made available for commercial production in 1935 after being evaluated as a new citrus hybrid. In 1940, Punjab Agriculture College and Research Institute, Faisalabad (Now the University of Agriculture, Faisalabad), Pakistan, introduced Kinnow in the Central Northern Punjab, Pakistan. The Kinnow-mandarin has a distinctive flavor that sets it apart from other related mandarins grown around the world attributable to the soil and climatic conditions in Central Northern Punjab. Due to the intrinsic good taste, the demand for Pakistan’s Kinnow increases in Pakistan as well as in foreign markets. As a result, the area under cultivation of Kinnow increases over time (2). In Pakistan, 85% of the citrus crop is of the Kinnow variety (*Citrus reticulata* Blanco). Pakistan is the source of almost 90% of the world’s production of this cultivar and 97 % of total citrus production comes from Punjab province. Sargodha, alongside Mandi Bahauddin and Khushab, two of its neighboring districts are known as the primary citrus growing regions in Central Northern Punjab. According to areas covered by various varieties, the Kinnow variety accounts for roughly 85% of the citrus, the other includes Musambi having 10%, Feutral with 4%, and Blood Red I% of citrus varieties produced (2).

The Pakistani citrus is preferred for its low price in the international markets. The competition in international market is much higher for Pakistani citrus. The problems faced by exporters include disease of the product and low research in the citrus improvement that does not meet the demand of importers (3). Pakistan exports citrus fruits to various markets, and many new exporters enter these same markets, competing with more established rivals. This competition has led to pressure on export prices, as many exporters compete primarily on price in the absence of distinct market positioning or product differentiation.

As a result, many exporters experience losses due to thin profit margins, unanticipated supply chain issues, or quality discounts demanded by importers due to subpar product quality upon arrival. Price competition among exporters has shifted bargaining power to importers. When exporters compete in a market without coordination, all exporters suffer, as one Pakistani citrus supplier is always willing to offer a lower price than others, regardless of demand in the target country. Notably, the average unit price for Pakistani mandarins is only 44% of the global average. This low unit price, which negatively impacts the profitability of the export value chain, is largely attributed to unhealthy price competition (4).

Similar to this, Pakistan’s citrus exporting sector faces a number of difficulties, including poor quality produce (only less than 30% of the nation’s total citrus production), particularly too many seeds in the fruit and presence of citrus canker, reliance of the farmers on a single variety for a brief period of time, dependence on a small number of markets, non-compliance with SPS protocols, and a lack of certifications. Despite being the largest citrus exporter in the world, mandarins have so far had little success entering developed nation markets because of their high seed content, poor quality, and quarantine requirements. In some export markets, seedless tangerines, oranges, mandarins, and clementine from China, Turkey, and Morocco are replacing Pakistani citrus as easy peelers.

Consequently, Pakistani citrus often receives less popularity than its competing products. One of the major obstacles to the citrus fruits, export from Pakistan is itself the fruit quality. It is evident that a higher proportion of first-grade fruit would be required if citrus was to become more competitive on the global market. Pakistan citrus earns one of the lowest export prices on the global market because to its poor quality (5). Despite all of these issues, Pakistan has significantly increased the value of its citrus exports. Pakistan is currently one of the top 20 producers of citrus in the world. In the past, the top export destinations for Pakistani citrus products were Afghanistan, Indonesia, United Arab Emirates, Philippines, Sri Lanka, Saudi Arabia, and Singapore. However, in 2021 the trend has now been changed as Afghanistan, Russian Federation, Philippines, Indonesia, and United Arab Emirates have become the largest market for Pakistani citrus exports.

Numerous scholars have conducted research on citrus exports because of how crucial they are to Pakistan’s economy. Haleem et al. (1) briefly investigated the Pakistan’s export supply function for citrus fruit. Co-integration and error correction techniques were used in the study’s empirical analysis of time series data from 1975 to 2004. The study’s findings indicate that although domestic pricing and domestic production have a negative impact on the country’s citrus exports, whereas export price, exchange rate, and GDP had a favorable impact. Also, after 9 years, Ayesha (6) examined the trends of quantity and value in Pakistan’s citrus export. The quadratic trend model was used in the study, which employed time series data from 1990 to 2011. The research estimated citrus export volume and value for the years 2012 to 2016. The study’s anticipated export quantities and values were quite accurate and demonstrated rising trends in Pakistan. However, Ahmad et al. (7) analyzed the whole citrus value chain and determined the key variables influencing the export of citrus from Pakistan. Value chain mapping, regression analysis, and data from surveys and interviews were all employed in the study. According to the study, large, well-established exporters with a high export volume and poor margin business strategy dominate the citrus export market. Citrus exports from Pakistan are mostly made to low or middle-income nations since exporters lack the skills necessary to compete in the developed economies due to the strict quality standards imposed by the Sanitary and Phytosanitary Agreement of the WTO. Regression analysis revealed that marketing research and quality certification had a strong influence on Pakistani citrus exports.

Also, many researchers, from citrus exporting countries have also conducted their studies on citrus exports in their countries. For the period of 2007–2012, Ozer & Koksal (8) employed a gravity model to Turkey citrus exports and examined the main variables affecting its citrus exports to major trade partners. The study’s findings suggested that crucial factors influencing citrus exports include international accords like the Black Sea Economic Cooperation agreement and the commercial cooperation between the European Union and Turkey. Citrus exports are negatively affected by transportation expenses. Furthermore, it is estimated that appreciation in the Turkey’s real exchange rate benefits the low-price citrus-producing nations. Bakari (9) examined the impact of citrus exports on Tunisia’s economic growth using yearly data from 1970 to 2016. Co-integration analysis with an error correction model was used to obtain empirical estimates. The results showed that citrus exports do not have long-term impact on economic growth. However, there is a short-term positive unidirectional causal relationship between citrus exports and economic growth.

Furthermore, several researchers have included trade agreement variables in the gravity model to analyze the impact of trade agreements on the export performance of Pakistan and other exporting countries. Cardamone (10) assessed the impact of preferential trade agreements on European imports of fresh grapes, pears, apples, oranges, and mandarins over the period 2001–2004 using the gravity model. The study’s results indicated that the GSP scheme appears effective in increasing EU imports of apples and mandarins, while the Cotonou Agreement successfully boosts EU imports of fresh grapes and mandarins. Additionally, regional trade agreements seem to be effective in expanding EU imports of all fruits except oranges from eligible countries. Ehsan (11) examined the impact of the free trade agreement (FTA) on the trade patterns of goods between Pakistan and China. The study used time-series data from 2003 to 2010 and employed a gravity model for empirical estimations. Results from the gravity model suggested that the Pak-China FTA does not affect the bilateral trade flow from Pakistan to China or from China to Pakistan. However, China’s GDP has a positive influence on Pakistan’s exports to China, whereas Pakistan’s GDP does not have much influence on its exports to China. Tayyab & Ali (12) investigated the determinants of Pakistan’s exports to selected Asian economies using a gravity model of trade. The study utilized panel data covering the period from 1980 to 2020 and employed pooled OLS, fixed effects, and random effects estimation techniques. The results showed that the product of Pakistan’s GDP and the GDP of selected Asian countries positively and significantly impacts Pakistan’s exports. Similarly, the product of Pakistan’s *per capita* income and the *per capita* income of selected Asian economies also positively impacts Pakistan’s exports. The impact of distance is negative but insignificant. Additionally, the study found that language and free trade agreements (FTAs) have an insignificant impact on exports. Jabbin et al. (13) examined the effects of the South Asian Free Trade Agreement (SAFTA) on the exports of Pakistan during the period 2006–2016 using the gravity model. In this study, SAFTA’s effect is measured by the difference between most favored nation (MFN) and preferential tariff rates. The results of the study suggested that SAFTA has a negative effect on the total exports of Pakistan, as the coefficient of SAFTA is negative. Moreover, the GDP of export destination partners positively affects Pakistan’s exports, while Pakistan’s own GDP also has a positive effect on its exports. The coefficient associated with distance is negative and statistically significant. The population coefficient of export destination partners is statistically significant and positive. The coefficients associated with common borders and common languages are negative and positive, respectively, both of which are statistically significant at the 1 percent level.

The analysis of the aforementioned studies leads us to the conclusion that previous research on citrus exports in Pakistan concentrated on the export supply function, trends in export quantity and value, the citrus value chain, and factors affecting the export of citrus from the nation at the firm level. We rarely found a research in Pakistan that examined the cross-border variables effecting citrus exports from the nations while analyzing the export demand function from Pakistan. Internationally, Ozer & Koksal (8) focused on the cross-boundary factors that affect Turkey’s citrus exports whereas Bakari (9) bridged a proper link between citrus exports and economic growth. However, the first study used relatively older panel estimation techniques like fixed effect and random effect whereas the second study employed time-series data. Similarly, past studies on the impact of trade agreements, such as free trade agreements, preferential trade agreements, and SAFTA, on exports have generally considered the overall exports of the country. Cardamone (10) is the only researcher whose analysis focused specifically on fruits. However, there is a scarcity of research in Pakistan examining the effect of trade agreement variables on citrus exports. Therefore, this study estimated export demand function for Pakistani citrus by considering the cross-border factors that affect exports of citrus from the country, since it is never been treated before. To achieve this objective, the study employed a gravity model using panel data from the top 22 countries to which Pakistan exports citrus products. The gravity model is a valuable econometric method for describing trade and is currently widely used in panel data trade studies (8). This study is the first large-scale investigation from Pakistan to employ a gravity model, specifically a two-way fixed effect panel data approach, to analyze the determinants of citrus export. This innovative methodological approach provides robust insights and contributes to the existing body of knowledge on international trade and export dynamics.

Citrus fruits are a vital component of Pakistan’s agricultural exports. However, the country faces challenges in maintaining and enhancing its competitiveness in the global market. Understanding the determinants of export performance is crucial for devising strategies that can improve the efficiency and profitability of citrus exports. Similarly, enhancing the competitiveness of citrus exports can lead to increased foreign exchange earnings, economic growth, and improved livelihoods for those involved in the citrus supply chain. Furthermore, the international trade landscape is continuously evolving, with changing the status of trade agreements. Understanding how these agreements affect Pakistan’s citrus exports can help stakeholders adapt to market changes and seize new opportunities. By analyzing the export dynamics with Pakistan’s top 22 trading partners, the study aims to strengthen existing trade relationships and identify potential areas for expanding market access. This can lead to more robust and diversified trade partnerships, benefiting the overall economy. The results of this study will provide valued input and feedback for the policymakers, enabling them to formulate appropriate commercial policies for citrus products exports. The results of the study on the factors influencing export markets will undoubtedly assist citrus exporters in Pakistan.

## 2 Materials and methods

### 2.1 Data

In this study, we used a panel dataset of 22 countries to whom Pakistan exports its citrus, the data covers the period from 2003 to 2021. The required data was obtained from the GOP (14, 15), World Bank (16), UN Comtrade Database (17), and google maps. According to UN Comtrade Database (17) Pakistan exports citrus to around 48 countries in the time duration 2003–2021. However, 22 countries are found to whom Pakistan exports its citrus regularly throughout the study period whereas 26 countries get Pakistani citrus on non-regular bases. Therefore, in this study we consider those 22 countries to whom Pakistani export it citrus on regular basis. Names of these countries are given in Table 1. Since the data on the exports volume of the citrus from Pakistan to 22 countries are available from 2003 to 2021; therefore, we construct our dataset from 2003 to 2021. The UN Comtrade Database (17) have data on the citrus export net weight (kg) and citrus export trade value (US$) for Pakistan to 22 trading partner countries across the globe. However, this dataset did not provide any information on the price of citrus exports in the importing countries. Therefore, we estimate the price of citrus in the importing country by dividing the citrus export trade value (USD) by citrus export net weight (kg). We collected data for the real *per capita* income, gross domestic product (GDP), population, and exchange rate from the World Bank (16). Furthermore, the data for the trade agreements are compiled from GOP (14, 15). Finally, the data on the distance between Pakistan’s capital and its trade partners’ capitals is taken from Google Maps.

**Table 1:** Region-wise list of countries to which Pakistan exports citrus.

Region	Countries in the region	Number of countries in the region
South Asia	Afghanistan, Bangladesh, Maldives, Sri Lanka	4
Central Asia	Azerbaijan	1
Middle East	Bahrain, Kuwait, Oman, Qatar, Saudi Arabia, United Arab Emirates	6
North America	Canada	1
East Asia	China: Hong Kong SAR	1
South East Asia	Indonesia, Malaysia, Philippine, Singapore, Viet Nam	5
Africa	Mauritius	1
Eastern Europe	Russian Federation, Ukraine	2
North Eastern Europe	Lithuanian	1
Total Number of countries		22

Source: World Bank (16) and UN Comtrade Database (17).

### 2.2 Model and estimation methods

The standard gravity model for international commerce is one method that might be used to model the cross-border factors that influence citrus exports from Pakistan. The most basic version of the gravity model for international commerce demonstrates a positive relationship between any two trading partners’ export volumes and their national incomes and a negative relationship between their export volumes with the distance between them (18). We follow Geraci and Wilfreid (19), Prewo (20) Pöyhönen (21), and Tinbergen (22) and specify the following gravity model:


(1) $$X_{ijt}=\frac{Y_{jt}}{A_{ijt}}{\left(\frac{D_{ijt}}{P_{jt}}\right)}^{1-\sigma }$$

where $X_{ijt}$ is the volume of citrus exports from country i (Pakistan) to country j (importing country) in year t , $Y_{jt}$ are the US$ value of *per capita* GDP in country j for year t , $D_{ijt}$ is the distance between country i and country j in year t , $P_{jt}$ is the price of exporting citrus in country j in year t , $A_{ijt}$ are the other explanatory variables which either restricts or promote the citrus exports among country i and country j , and σ is the elasticity of substitution between all fruits. The log-linear form of the gravity model of equation (1) is given by:


(2) $$\begin{array}{l} \ln\left(X_{ijt}\right)=\ln\left(Y_{jt}\right)+\left(1-\sigma \right)\ln\left(D_{ijt}\right)-\left(1-\sigma \right)\\ \;\ln\left(P_{jt}\right)-\ln\left(A_{ijt}\right) \end{array}$$

Following Bergstrand (23), Egger and Pfaffermayr (24), Lewer and Van den Berg (25) and Narayan and Nguyen (26), we employ two explanatory variables $\left(A_{ijt}\right)$ such as exchange rate between country i and country j $\left(ER_{ijt}\right)$ and population size in country j $\left(POP_{jt}\right)$ , Similarly, following Ozer & Koksal (8), we employ the set of trade agreements and regional dummies ( $DTA_{i},DR_{i}$ ). By adding the parameters and error term to equation (2) and by ignoring the negative signs of the price $\left(P_{jt}\right)$ , population size $\left(POP_{jt}\right)$ , and exchange rate $\left(ER_{ijt}\right)$ , it gives the following econometric specification of the model:


(3) $$\begin{array}{l} \ln\left(X_{ijt}\right)=\alpha_{i}+\gamma_{j}+\lambda_{t}+\alpha_{1}\ln\left(Y_{jt}\right)+\alpha_{2}\left(D_{ijt}\right)\\ \;+\alpha_{3}\left(P_{jt}\right)\;+\alpha_{4}\ln\left(POP_{jt}\right)+\alpha_{5}\left(ER_{ijt}\right)\\ \;+\alpha_{6}\left(DTA_{i}\right)+\alpha_{7}\left(DR_{i}\right)+\varepsilon_{ijt} \end{array}$$

where $\alpha_{i}$ is the domestic country effect, i=1 , $\gamma_{j}$ is the partner country effect, j=1,….,24 and $\lambda_{t}$ is the time effect, t=1,…,12 , and $\varepsilon_{ijt}$ is the error term. Equation (3) presents the generalized form of the two-way fixed effect panel data model. In our analysis, firstly, we considered both random and fixed effect models. However, later on, we used Hausman test. The Hausman test can be used to compares the random and fixed effect models and suggesting that appropriate model. Ultimately this will enhance the accuracy and reliability of our findings and contributing in investigating the relationship between variables. We have chosen to utilize the fixed effect model due to its acknowledged robustness in addressing the challenges posed by panel heterogeneity. In our research context, panel datasets are prevalent, comprising observations over time for multiple countries. These datasets often exhibit correlations among countries and time, along with variations in individual country and time effects. The fixed effect model is favored for its ability to control for unobserved heterogeneity. In this analysis, first, we consider both random and fixed effect models. However, later on, for comparisons of the random and fixed effect models and for selecting appropriate models among the two, we used Hausman test. Furthermore, the regression models employed in the analysis differ in terms of the type of regression technique used. Equation (3) is first estimates by pooled ordinary least squares (OLS) approach, which assumes that all countries are homogeneous and does not account for country-specific effects. In contrast, the subsequent models incorporate various fixed effects to address the issue of country heterogeneity. Keeping this point in mind, equation (3) is estimated via time fixed effect. The time fixed effects model controls for unobserved factors that vary over time but are constant across countries. Next equation (3) is estimated via country fixed effect. The country fixed effects model accounts for country-specific characteristics that remain unchanged over the study period. Finally, equation (3) is estimated via time and country fixed effect. The time and country fixed effects model captures both the temporal and cross-country heterogeneity in the data. By incorporating these fixed effects, the latter models provide a more nuanced understanding of the factors influencing the outcome variable, as they allow for the evaluation of country-specific impacts that are not captured by the pooled OLS approach.

We estimate the aforementioned model using time and country fixed effect regression with STATA to determine the effect of cross-border factors on the citrus exports from Pakistan. The dependent variable in our model is the amount of citrus that Pakistan exports to its 22 trading partners. The explanatory variables include, real *per capita* GDP, distance, export price of citrus, population, exchange rate, trade agreement dummies, and regional dummies which are described below:

Citrus exports: As a dependent variable in this study, we use the quantity of citrus exports (kg) from Pakistan to 22 trading partners (1, 8, 9). *Per capita* GDP: the *per capita* income in the trading partner countries has a significant and positive impact on the quantity of exports from the exporting country. As the *per capita* income in the trading partner countries increases, the demand for the exporting country’s products also rises, leading to a greater quantity of exports (24). For this purpose, we employ the real *per capita* GDP of the trading partner nations in US dollars as an explanatory variable (1, 8, 9, 27).

Distance: According to the gravity model, exporting is inversely correlated with distance, and nations that are physically close to one another will experience lower transportation and other costs than those that are farther apart. Therefore after the estimation the distance variable is anticipated to have either positive or negative values (8).

Export price of citrus: Economic research has identified a negative relationship between export volumes and pricing. When the price of the exported good increases in the exporting country, the exporting country loses its competitive advantage in the importing country’s market, leading the importing country to reduce its purchases of that commodity. Conversely, when the price of the exported goods rises in the importing country, the exporting country is able to increase its exports and earn higher export revenues. However, the consumer in the importing country has less buying power as a result of the increase in the price of exporting commodities. As a result, people switch their consumption from imported commodities to domestic alternatives. Once more, this diversification lowers the export earnings of the exporting country (28). We estimate the price of citrus in the importing country by dividing the citrus export trade value (USD) by the citrus export net weight (Kg) in order to account for the effect of citrus price in the importing country (1, 8, 9).

Population size: The need for imports is increased with a large population; as a result, the country imports a wide variety of commodities from exporting nations. This shows that population has a positive impact on bilateral trade (28). On the other hand, total GDP and GDP *per capita* are reliable indicators of import demand and export supply, suggesting that population has a negative influence on international trade. The population size is significant; therefore, we employ it as an explanatory variable (1, 8, 9).

Exchange rate: The exchange rate of a currency reflects the volatility in its value. When the exchange rate rises and the domestic currency appreciates, domestic products in the importing nation become less expensive as a result, and vice versa. The exchange rate is used as an explanatory variable in this study (1, 8, 9, 26).

Trade agreements: Trade agreements between exporting and importing countries can have a significant impact on the flow of exports and imports between those nations. Factors such as free trade agreements, preferential trade agreements, and regional trade pacts like the South Asia Free Trade Agreement (SAFTA) can influence the export dynamics, particularly for specific commodities like citrus fruits in the case of Pakistan and its trading partners. The details of these agreements and incentives provide for citrus exports in these agreements are given in Table 2. We introduce a separate dummy for each trade agreement and, if the exporting nation is a signatory to the agreement, we assign value 1; otherwise, we assign value 0 (8).

**Table 2:** List of trade agreements of Pakistan with countries to which Pakistan exports citrus.

Trade agreements	Incentives provide for citrus exports in the agreements
Free trade agreements:	
Pak-China Free Trade Agreement in Goods & Investment	According to the terms and conditions of this agreement Pakistan will get market access at zero duty on citrus fruit
Pak-Malaysia Free Trade Agreement	Under this agreement Malaysia provides market access to Pakistani oranges/kinnow at 5 percent base rate and without specific duty
Pak-Sri Lanka Free Trade Agreement	Under this agreement Pakistan, would gain duty free access on oranges in the Sri Lankan market.
Preferential trade agreements:	
Pak-Indonesia Preferential Trade Agreement	Under this agreement Indonesia provides market access to Pakistani oranges/kinnow/Mandarin/lemon at 0 percent import duty
Pak-Mauritius Preferential Trade Agreement	Under this agreement Mauritius reduced custom duty on Pakistani citrus from 50 percent to 15 percent
Agreement on South Asian Free Trade Area:	
SAFTA	Under this agreement Bangladesh, Maldives, and Sri Lanka reduces imports duties on Pakistani oranges and other citrus fruits
Other trade agreements:	
Pak-Afghanistan Transit Trade Agreement (PATTA)	The primary purpose of PATTA is to provide Afghanistan with access to international markets through Pakistani ports and to facilitate transit trade between the two countries. This agreement reduces transportation costs and transit times for Pakistani citrus exports, hence the citrus exports to Afghanistan increases.
Pak-Malaysia Early Harvest Program*	Under this agreement Malaysia provides tariff free market access to Pakistani citrus fruit (fresh or dried)
Pak-China Early Harvest Program	Under this agreement China provides tariff free market access to Pakistani citrus

Source: GOP (14, 15). *An Early Harvest Program (EHP) is not a full-fledged trade agreement but rather a preliminary or interim arrangement within the broader context of trade negotiations. It is designed to quickly implement benefits from trade liberalization on a selected list of goods or services before a comprehensive trade agreement is finalized.

Region: The adjacent importing countries provide the exporting country the chance to increase its product exports at lower costs. On the other hand, exports may be decreased by importing countries in distant regions. The 22 countries that Pakistan exports citrus to are spread over 10 distinct geographic areas. The details of regions, the name of countries in each region, and number of countries in each region are given in Table 1. We introduce nine dummies for a total of 10 areas, and we give each nation a value of 1 if it belongs to a certain region, and 0 otherwise (8).

## 3 Results and discussion

### 3.1 Analysis of Pakistan’s citrus exports trends

**Figure 1:**
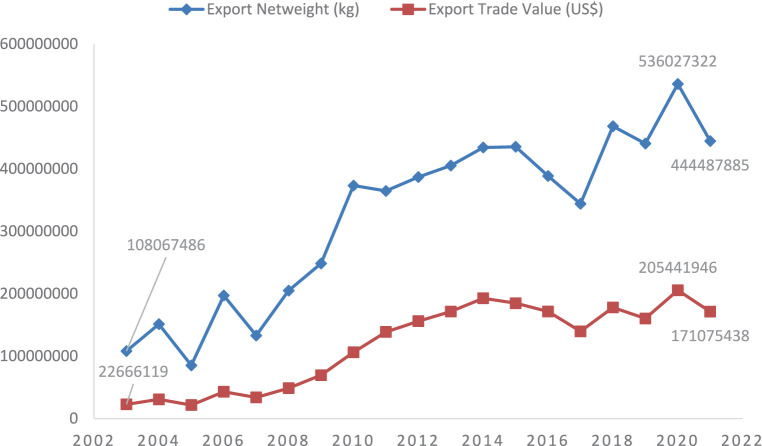
Exports of citrus from Pakistan. Source: Computed by authors based on data taken from UN Comtrade Database (17).

In the international market, Pakistan has expanded its citrus exports quite rapidly. Pakistan is now ranked among the top 20 citrus producing countries in the world. From 2003 to 2021, the country increased its citrus exports from 108,067,486 kg (108,067.486 tons) to 444,487,885 kg (444,487.885 tons) and increase the citrus export earnings from USD.22,666,119 (USD.22.7 million) to USD.171,075,438 (USD.171.1 million) as shown in Figure 1. This shows that the country had realized an increase of 24% in citrus exports quantity in the past 19 years whereas 13 percent increase in the export earnings from citrus. Despites the fact that Pakistani citrus earn 44 percent lower price as compared to the world unit price of citrus, such a reasonable increase in export revenue is a good sign. The country can further increase its export earnings by improving its citrus quality.

**Figure 2:**
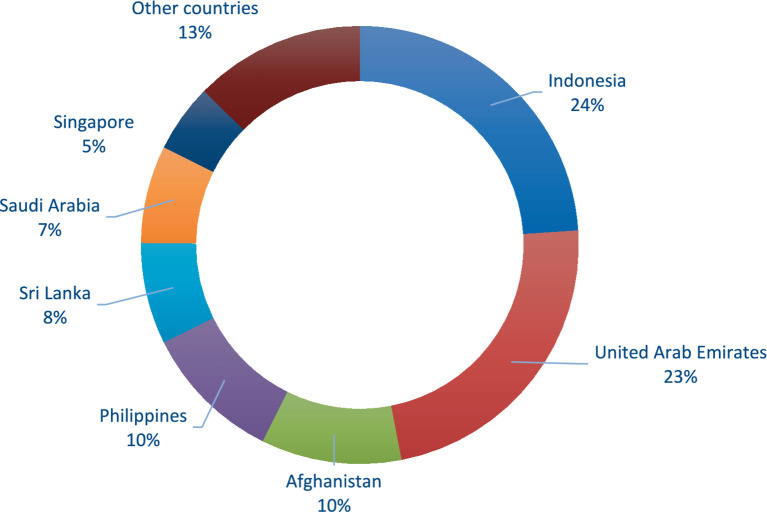
Top countries where Pakistan exports its citrus products in 2003. Source: Computed by authors based on data taken from UN Comtrade Database (17).

**Figure 3:**
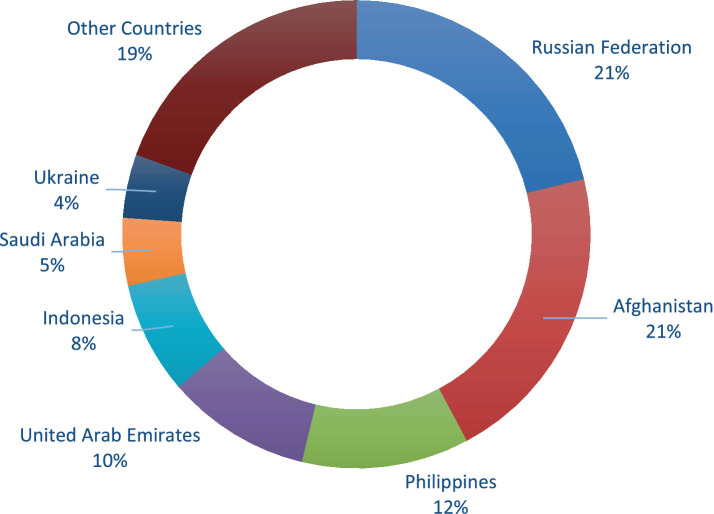
Top countries where Pakistan exports its citrus products in 2021. Source: Computed by authors based on data taken from UN Comtrade Database (17).

In 2003, Indonesia and United Arabs Emirates were the leading markets for Pakistani citrus exports for the fact that 47% of the citrus exports were made to these countries from Pakistan. The remaining 53 percent citrus export from Pakistan were made to Afghanistan, Philippine, Sri Lanka, Saudi Arabia, Singapore, and to other countries as shown in Figure 2. However, in 2021, the trend is changed and Afghanistan and Russian Federation are become the largest market for Pakistani citrus exports and about 42 percent of the citrus exports from Pakistan goes to these countries. Figure 3 shows that the remaining 58 percent citrus exports from Pakistan goes to Philippines, Indonesia, United Arab Emirates, and other countries. There are several possible reasons for the shift from Indonesian and United Arab Emirates markets to Afghanistan and Russian Federation markets for Pakistani citrus exports. The close geographic proximity and strong trade bonds between Pakistan and Afghanistan have facilitated easier and more cost-effective transport of citrus products. Similarly, the political, cultural, and economic interdependency between the two nations leads to favorable trade policies and reduced tariffs. The recent developments in transport and logistics infrastructure between Pakistan and Afghanistan, significantly reduce transit times and costs, making it more attractive to export there. Moreover, the increasing diplomatic and economic relations between Pakistan and Russian Federation after the Russian-Afghan war have led to increased trade. Pakistan has been actively seeking to diversify its export markets, and Russia’s large population and demand for fresh produce make it an attractive market. Furthermore, the raise in the cost of production and transportation of citrus fruit might have made it more economical to export to Afghanistan and Russian Federation compared to Indonesia and the UAE. Besides, the increasing competition in traditional markets for Pakistani citrus like Indonesia and the UAE from other citrus-exporting countries also pushed Pakistani citrus exporters to seek new citrus markets where they have a competitive edge. Similarly, a huge number of Pakistani migrants in the United Arab Emirates (UAE) demanded Pakistani citrus thus citrus exports to UAE also increase. Other countries, like Hong Kong (China), Malaysia, Sri Lanka, Indonesia, Bangladesh, and Maldives are partner with Pakistan and are signatory of the free trade agreements, preferential trade agreements, and SAFTA, respectively. Therefore, citrus exports from Pakistan to these countries also increases over time.

### 3.2 Descriptive statistics

Table 3 provides a summary of the key variables, both dependent and independent, used in this study. It is observed that on average Pakistan export 1,350 tons of citrus annually to each partner country included in the sample. This is a significant amount of citrus export. This show that due to the inherent good quality and taste, the demand for Pakistani citrus is still high in foreign markets. We have witnessed the average *per capita* income in the partner countries to be USD.17,941. The data suggests that Pakistan’s citrus exports are primarily directed toward high-income countries.

**Table 3:** Descriptive statistics.

Variables	Definition	Mean	Standard deviation
Dependent:			
Quantity of citrus exports	Quantity of citrus exports from Pakistan to 22 trading partner countries (kg)	13,500,000	31,300,000
Explanatory:			
Income:			
*Per capita* income	*Per capita* income (constant USD) in 22 trading partner countries	17,941	18,166
Distance:			
Distance	Distance between Pakistan’s capital and its 22 trading partners’ capital (Km)	3,646.3	2,080.6
Citrus price:			
Price	Price of Pakistani citrus in 22 trading partner countries (USD/kg)	0.35	0.12
Population:			
Population	Total population in 22 trading partner countries	43,900,000	63,200,000
Exchange rate:			
Exchange rate	Exchange rate in 22 trading partner countries (relative to USD)	1,420.1	4,670.5
Trade agreements:			
No agreement (Reference)			
Free trade agreement (Yes = 1, No = 0)	1 if free trade agreement is signed between Pakistan and citrus importing country and 0, otherwise	0.14	0.34
Preferential trade agreement (Yes = 1, No = 0)	1 if preferential trade agreement is signed between Pakistan and citrus importing country and 0, otherwise	0.09	0.28
SAFTA (Yes = 1, No = 0)	1 if SAFTA is signed between Pakistan and citrus importing country and 0, otherwise	0.14	0.34
Region:			
Other region (Reference)			
South Asia (Yes = 1, No = 0)	1 if the citrus importing country located in South Asia and 0, otherwise	0.18	0.38
Central Asia (Yes = 1, No = 0)	1 if the citrus importing country located in Central Asia and 0, otherwise	0.045	0.21
Middle East (Yes = 1, No = 0)	1 if the citrus importing country located Middle East and 0, otherwise	0.27	0.44
North America (Yes = 1, No = 0)	1 if the citrus importing country located in North America and 0, otherwise	0.045	0.21
East Asia (Yes = 1, No = 0)	1 if the citrus importing country located in East Asia and 0, otherwise	0.045	0.21
Southeast Asia (Yes = 1, No = 0)	1 if the citrus importing country located in Southeast Asia and 0, otherwise	0.23	0.42
Africa (Yes = 1, No = 0)	1 if the citrus importing country located in Africa and 0, otherwise	0.045	0.21
Eastern Europe (Yes = 1, No = 0)	1 if the citrus importing country located in Eastern Europe and 0, otherwise	0.09	0.28
Northeastern Europe (Yes = 1, No = 0)	1 if the citrus importing country located in Northeastern Europe and 0, otherwise	0.045	0.21
Observations		418	

Source: Computed by authors based on panel from 22 trading partners of Pakistan.

The data analysis reveals numerous key insights about Pakistan’s citrus export landscape. Firstly, Pakistan’s citrus exports are primarily directed toward high-income countries, with an average distance of 3,646 km from the exporting country. This suggests that Pakistan’s citrus products reach not only neighboring markets but also to the distant countries as, indicating a diversified export portfolio. The average price received by Pakistan for its citrus exports is relatively low, at around $0.35 per kilogram. This is comparatively below the prices obtained by other major citrus exporting countries, such as Brazil, the United States, the European Union, Egypt, China, Turkey, and Morocco.

The average population of Pakistan’s trading partner countries is estimated at 43.9 million, implying that Pakistan targets markets with a significant consumer base for its citrus products. Additionally, the data indicates that Pakistan’s citrus exports are often directed toward countries with depreciated currencies, with an average exchange rate of 1,420 relative to the US dollar. Regarding trade agreements, the analysis reveals that 28% of Pakistan’s citrus exports go to countries with which it has signed free trade agreements, including the South Asia Free Trade Agreement (SAFTA). Furthermore, 9% of Pakistan’s citrus exports are destined for countries with which it has established preferential trade agreements.

These findings provide valuable insights into the dynamics of Pakistan’s citrus export sector, highlighting the importance of factors such as market size, exchange rates, and trade agreements in shaping the country’s export performance. The remaining 63 percent of citrus exports are going to those countries to whom Pakistan has still not sign any trade agreement. Comparing the regions, it is observed that Pakistan exports 68 percent of its citrus to the countries located in Middle East, Southeast Asia, and South Asia. The remaining 32 percent exports are diverted to the countries located in other regions. This show that Middle East, Southeast Asia, and South Asia are the biggest citrus markets for Pakistan. Citrus markets in other regions like Central Asia, North America, East Asia, Africa, and Europe are still open for Pakistani citrus, Pakistan can increase its exports to these market in the future.

### 3.3 Results of gravity model for citrus exports

Table 4 presents four different regression models that examine the relationship between the quantity of citrus exports from Pakistan and various independent variables. The independent variables are grouped into five categories: income, distance, citrus price, population, and trade agreements. The income category includes the *per capita* income of each country that import citrus from Pakistan, while the distance category includes the distance between Pakistan and the citrus importing countries. The citrus price category includes the price of Pakistani citrus fruit in each importing country, and the population category includes the population of each citrus importing country. Finally, the trade agreements category includes three variables that represent the existence of different types of trade agreements between Pakistan and the citrus importing countries.

**Table 4:** Results of gravity model for citrus exports.

Model:	(1)	(2)	(3)	(4)
Dependent variable: Quantity of citrus exports (ln)	Pooled OLS	Time fixed effect	Country fixed effect	Time & country fixed effect
Income:				
*Per capita* income (ln)	0.677***	0.572***	1.285***	0.777
	(0.125)	(0.125)	(0.347)	(0.496)
Distance:				
Distance	0.00110***	0.00117***	−0.00406***	−0.00149
	(0.000209)	(0.000206)	(0.00133)	(0.00217)
Citrus price:				
Price	0.0156	−0.352***	−0.0129	−0.0738
	(0.0579)	(0.0973)	(0.0631)	(0.0888)
Population:				
Population (ln)	1.017***	0.901***	2.222***	1.610***
	(0.0674)	(0.0701)	(0.338)	(0.521)
Exchange rate:				
Exchange rate	−0.000115***	−0.000115***	0.000016	0.000008
	(0.00002)	(0.00002)	(0.00007)	(0.000075)
Trade agreements:				
No agreement (Reference)				
Free trade agreement (Yes = 1, No = 0)	0.653**	0.627**	3.200**	2.761*
	(0.256)	(0.251)	(1.449)	(1.502)
Preferential trade agreement (Yes = 1, No = 0)	0.555	0.627	7.937***	4.849*
	(0.425)	(0.418)	(1.950)	(2.881)
SAFTA (Yes = 1, No = 0)	−7.774***	−7.681***	8.340**	0.626
	(0.607)	(0.596)	(4.028)	(6.466)
Region:				
Other region (Reference)				
South Asia (Yes = 1, No = 0)	9.617***	9.141***	−5.690	2.579
	(1.646)	(1.636)	(4.648)	(7.167)
Central Asia (Yes = 1, No = 0)	1.137	0.433	−1.763	0.744
	(1.376)	(1.381)	(2.270)	(2.819)
Middle East (Yes = 1, No = 0)	4.567***	4.153***	−4.062	2.398
	(1.408)	(1.406)	(3.919)	(5.837)
North America (Yes = 1, No = 0)	−8.899***	−9.182***	24.03***	9.294
	(1.732)	(1.721)	(7.991)	(12.72)
East Asia (Yes = 1, No = 0)	−0.961	−1.112	−5.205**	−2.343
	(1.342)	(1.340)	(2.056)	(2.854)
Southeast Asia (Yes = 1, No = 0)	−0.699	−1.015	−0.766	−0.878
	(1.252)	(1.255)	(1.073)	(1.105)
Africa (Yes = 1, No = 0)	0.411	−0.205	7.231***	4.520
	(1.365)	(1.368)	(2.117)	(2.774)
Eastern Europe (Yes = 1, No = 0)	1.666	1.593	2.347	3.195*
	(1.299)	(1.299)	(1.752)	(1.849)
Northeastern Europe (Yes = 1, No = 0)	0.140	−0.399	6.401***	4.613*
	(1.305)	(1.309)	(2.058)	(2.410)
	No	Time	No	Time
	No	No	Country	Country
Constant	−13.88***	−10.56***	−20.60***	−16.61***
	(2.642)	(2.698)	(4.303)	(5.061)
Diagnostic check:				
Observations	418	418	418	418
R-squared	0.704	0.728	0.799	0.806
F-statistics	56.1***	29.2***	59.9***	35.2***
Root MSE	1.24	1.22	1.03	1.04
Hausman Test		12.68*	15.66**	22.07***

Source: Computed by authors based on panel from 22 trading partners of Pakistan. Standard errors in parentheses, ***p < 0.01, **p < 0.05, *p < 0.1.

Since the models differ in terms of the type of regression analysis used such that pooled OLS (model 1), time fixed effect (model 2), country fixed effect (model 3), and time and country fixed effect (model 4). However, the pooled OLS’s fundamental assumption is that it does not account for country heterogeneity. OLS assume that all countries are homogeneous and does not evaluate country-specific impacts. On the other hand, the country and time fixed effects adds heterogeneity to the model. Thus, heterogeneity of the countries in the model is taken into account by time fixed effect, country fixed effect, and the time & country fixed effects. Our main target is to highlight and interpret the results of the second and third regressions with time and country fixed effects given in Table 1, model (2) and model (3). Therefore, we interpret the results of these regressions.

The last panel of Table 4, reports the results of some diagnostic checks, such as the number of observations, R-squared, F-statistics, root mean square error (RMSE), and Hausman test. These checks help to assess the overall goodness of fit of the regression model and the validity of the estimated coefficients. The R-squared values of the regression models range from 0.70 to 0.81, indicating that the explanatory variables account for 70 to 81 percent of the variation in citrus exports. This suggests a strong explanatory power of the models. Furthermore, the statistically significant F-test results allow us to reject the null hypothesis that the explanatory variables do not bring about any change in citrus exports. This provides confidence in the overall significance of the model specifications. Importantly, the root-mean-squared error (RMSE) values, which range from 1.03 to 1.24, demonstrate that the models have been estimated with a high degree of precision, with relatively low levels of error. This further underscores the robustness of the modeling approach and the reliability of the insights derived from the analysis. On the basis of statistically significant values of the Hausman test for model (2), model (3), and model (4), we reject the null hypothesis that random effects models are consistent and efficient compared to fixed effects models. Thus, we concluded that the fixed effect models are more appropriate than random effect models. Therefore, we have proceeds with fixed effect models.

Table 4 presents the coefficient estimates for each independent variable, along with associated standard errors in parenthesis. The influence on the dependent variable is captured by the coefficient of the dependent variables. A statistically significant coefficient (indicated by the asterisks) suggests that the variable is associated with a statistically significant change in the dependent variable, considering other variables constant. Overall, the results from model (2) and model (3) suggests that *per capita* income, population, trade agreements and some regional dummies are positively associated with the quantity of citrus exports, while citrus price, distance, exchange rate, and other regional factors are negatively associated with citrus exports.

The findings from model 2 indicate a positive and statistically significant relationship between *per capita* income in the importing countries and Pakistan’s citrus exports. Specifically, the results suggest that a 1 percentage point increase in *per capita* income in the importing countries is associated with a 57-percentage point increase in citrus exports from Pakistan. This result aligns with the theoretical foundations of the gravity model, which posits that the demand for a country’s exports is positively influenced by the income levels of the importing markets. In other words, as the *per capita* income in the foreign markets rises, the demand for Pakistani citrus products tends to increase, all else being equal. From a practical standpoint, this implies that larger economies, in terms of GDP, are likely to import more citrus products from Pakistan. This underscores the importance of targeting high-income markets and understanding the role of economic size and purchasing power in driving export demand for Pakistani citrus. This result is consistent with findings of previous studies (1, 5, 8, 29).

The distance variable in model (3) is also statistically significant, but the relationship is negative, indicating that 1 kilometer (km) increase in distances between Pakistan and trading partners lead to 0.41 percent points decrease in citrus exports. The gravity model has described that geographically close countries will decrease transportation and other expenses more than with distant countries and that there is an opposite direction relationship between exports and distance. Thus the gravity model demonstrates a negative relationship among the volume of citrus exports between Pakistan and trading partners and the distance between them. This result is in accordance with the results of Ozer & Koksal (8), they found that geographical distances significantly affect citrus exports.

The citrus price variable in model (2) shows a statistically significant negative coefficient. This suggests that a one unit (USD) raise in the Pakistani citrus price in importing countries reduce citrus exports from Pakistan by 35 percent points. The Pakistani citrus is preferred for its low price in the international markets. Thus, any increase in the price of Pakistani citrus reduce its demand in the importing countries. This result is consistent with findings of Haleem et al. (1) whereas the result is in contradiction with findings of Ghafoor et al. (29).

The population variable also exhibits a positive and statistically significant association with citrus exports. The results suggest that a 1 percentage point increase in the population of the importing countries is linked to a 90-percentage point increase in citrus exports from Pakistan. This indicates that larger population centers tend to generate greater demand for Pakistani citrus products.

Regarding the role of trade agreements, the findings from model 3 reveal a significant positive relationship between various trade pacts and Pakistan’s citrus exports. Specifically, the results suggest that countries that have signed free trade agreements, preferential trade agreements, and the South Asian Free Trade Agreement (SAFTA) with Pakistan receive 3.2, 8.0, and 8.3 times higher citrus exports from Pakistan, respectively, compared to countries without any such trade agreements (14, 15).

The significance of these trade agreements is further highlighted in model 3. The results show that the countries that have signed free trade agreements and preferential trade agreements with Pakistan, as well as those that are signatories to SAFTA, received 3.2, 8.0, and 8.3 times higher citrus exports from Pakistan, respectively, compared to countries without any such trade agreements. This underscores the importance of economic cooperation and trade liberalization initiatives in facilitating the expansion of Pakistan’s citrus exports (14, 15).

These findings underscore the importance of economic cooperation and trade liberalization initiatives in facilitating the expansion of Pakistan’s citrus exports. The duty-free market access, tariff reductions, and improved trading environments established through these agreements have likely contributed to the growth of Pakistan’s citrus trade with its partner countries. However, there are still opportunities for the Government of Pakistan to explore and sign additional free trade agreements or preferential trade agreements with other major citrus importing countries, such as Azerbaijan, Bahrain, Canada, Kuwait, Lithuania, Oman, the Philippines, Qatar, Singapore, Ukraine, the Russian Federation, Saudi Arabia, the UAE, and Vietnam, to further expand the export markets for Pakistani citrus products. Previous research by Ozer and Koksal (8) has also found similar results when analyzing the primary factors that influence Turkey’s citrus exports to its major trading partners.

However, comparing these results with the results of Figures 2, 3, it is found that for some countries like Malaysia and Sri Lanka, which have signed free trade agreements with Pakistan, citrus exports from Pakistan to these countries have declined over time. The main reason for this, as described by the World Bank (30), is that while free trade agreements provide an initial boost to citrus exports from Pakistan, however; the long-term benefits from trade agreements are contingent on various factors such as consistent quality of the citrus fruit, market conditions, and competitiveness. The initial increase in citrus exports after signing a free trade agreement and the subsequent gradual decline over time, despite the trade agreement, are primarily because Pakistani citrus exporters did not effectively address these issues. Addressing these challenges in citrus export markets requires continuous improvements in quality, competitiveness, and addressing logistical and policy-related barriers.

Finally, the analysis also reveals the varying effects of regional factors on Pakistan’s citrus exports. In models 2 and 3, the coefficients for South Asia, the Middle East, North America, Africa, and Northeastern Europe were found to be positive and statistically significant. This suggests that countries located in these regions received 9, 4, 24, 7, and 6 times higher citrus exports from Pakistan, respectively, compared to countries in other regions (10, 11). In contrast, the coefficient for East Asia was negative and significant, indicating that countries in this region received 5 times lower citrus exports from Pakistan compared to other regions (10, 11). These regional differences may be attributed to the relative profitability of the citrus markets in the respective regions for Pakistani exporters. The findings are consistent with the results reported by Ozer and Koksal (8), who used different regional groupings, such as the European Union and the Black Sea Economic Cooperation, in their analysis of Turkey’s citrus exports.

These regional dynamics underscore the importance of understanding the nuances of international trade patterns and the unique characteristics of different export markets. Pakistani policymakers and exporters should consider these regional factors when formulating strategies to optimize the country’s citrus export performance and capitalize on the most lucrative market opportunities.

## 4 Conclusion and recommendations

Using panel data from the top 22 countries where Pakistan exports its citrus products using a gravity model, this study examined the cross-border factors that influence citrus exports from Pakistan. According to the gravity model, citrus exports in Pakistan increase as *per capita* income increase in the importing nations. This implies that Pakistani citrus exporters need to enhance the percentage of their exports going to high-income markets. However, despite being the largest citrus exporter in the world, Pakistani citrus exporters have only recently begun to enter markets in high-income nations. This may be because of their significant seed content, poor fruit quality, and strict quarantine laws for citrus fruit in high income countries. As a result, Pakistani citrus is typically given poor priority over other countries citrus. It is apparent that if the competitiveness of citrus in the high-income countries market is to be improved, Pakistani exports of first-grade citrus must be raised.

A rise in the price of Pakistani citrus in importing countries reduces Pakistani citrus exports. In international markets, Pakistani citrus is preferred due to its low pricing whereas other countries’ citrus is preferred in terms of quality. Furthermore, the citrus exporters from Pakistan faces increased competition in foreign markets. In the absence of product differentiation, Pakistani citrus exporters often end up competing primarily on pricing, which has put downward pressure on export prices in recent years. Low unit prices for Pakistani citrus and the ensuing low profitability of the exporters are said to be primarily caused by unfavorable pricing competition. Therefore, the citrus business in Pakistan can perform better in terms of exports by diversifying its citrus market from low-price nations like the UAE, Saudi Arabia, Afghanistan, and Russia to prospective high-end markets like France, the Netherlands, China, Hong Kong, and Indonesia.

The population variable is also positively associated with citrus exports, with statistically significant coefficient. The diversification of citrus market from low population countries like Afghanistan, Azerbaijan, Bahrain, Kuwait, Lithuania, Oman, Qatar, Singapore, UAE, and Viet Nam to potential high population markets like China, Indonesia, Bangladesh, and Malaysia can improve the export performance of Pakistan’s citrus industry. Existing import quantities in prospective high-end markets are sufficient for Pakistani citrus to establish itself. Because of its large market, free trade agreement status, closeness to Pakistan, and the CPEC project, China has great export potential. On the other hand, Indonesia provides zero-rated market access to citrus from Pakistan, leveling the playing field in the Indonesian market. Besides, Pakistan and Bangladesh are members SAFTA; thus, Pakistani exporters can easily increase its citrus exports share to Bangladesh with minimum tariff restrictions. Furthermore, Pak-Malaysia Free Trade Agreements offers a significant market access to Pakistani citrus exporters, offering an open field to citrus in the Malaysian market.

This study is a premier attempt to analyze the cross-border factors affecting citrus exports from Pakistan, and it yields excellent results. But we all know that nothing is flawless. This study also has several shortcomings that future researchers can address. To begin, this study merely calculated the export demand function for citrus produce. Future researchers can estimate the export demand function and export supply function for other fruits such as mango, apple, guava, dates, and peach etc. Second, this study was conducted on the citrus exports from Pakistan, keeping in mind that Pakistan is one of the world’s top citrus exporting countries. However, a large number of other nations, such as Spain, South Africa, China, the Netherlands, Egypt, South Africa, Morocco, Spain, Georgia, Uruguay, Greece, Lebanon, and Peru, are also included among the leading exporters of citrus. Future scholars can replicate this study on the exports of citrus from these nations.

## Data availability statement

The original contributions presented in the study are included in the article/supplementary material, further inquiries can be directed to the corresponding authors.

## Author contributions

NH: Conceptualization, Data curation, Project administration, Software, Validation, Visualization, Writing – review & editing. MN: Conceptualization, Writing – original draft, Writing – review & editing, Formal analysis, Investigation, Methodology, Project administration. GM: Conceptualization, Writing – original draft, Writing – review & editing, Funding acquisition, Software, Supervision. BA: Conceptualization, Data curation, Formal analysis, Funding acquisition, Investigation, Project administration, Resources, Writing – review & editing. AT: Formal analysis, Funding acquisition, Investigation, Methodology, Resources, Software, Validation, Visualization, Writing – original draft. AA: Conceptualization, Data curation, Formal analysis, Investigation, Methodology, Validation, Writing – original draft.
